# DSC and FTIR study on the interaction between pentacyclic triterpenoid lupeol and DPPC membrane

**DOI:** 10.1007/s10863-024-10030-1

**Published:** 2024-06-26

**Authors:** Cisem Altunayar-Unsalan

**Affiliations:** Ege University Graduate School of Natural and Applied Sciences, Bornova, Izmir 35100 Turkey

**Keywords:** Triterpenoids, Lupeol, Membrane interaction, DPPC, DSC, FTIR spectroscopy

## Abstract

Natural products are a great resource for physiologically active substances. It is widely recognized that a major percentage of current medications are derived from natural compounds or their synthetic analogues. Triterpenoids are widespread in nature and can prevent cancer formation and progression. Despite considerable interest in these triterpenoids, their interactions with lipid bilayers still need to be thoroughly investigated. The aim of this study is to examine the interactions of lupeol, a pentacyclic triterpenoid, with model membranes composed of 1,2‑dipalmitoyl‑sn‑glycerol‑3‑phosphocholine (DPPC) by using non-invasive techniques such as differential scanning calorimetry (DSC) and Fourier transform infrared (FTIR) spectroscopy. The DSC study demonstrated that the incorporation of lupeol into DPPC membranes shifts the L_β′_-to-P_β′_ and P_β′_-to-L_α_ phase transitions toward lower values, and a loss of main phase transition cooperativity is observed. The FTIR spectra indicated that the increasing concentration (10 mol%) of lupeol causes an increase in the molecular packing and membrane fluidity. In addition, it is found that lupeol’s OH group preferentially interacts with the head group region of the DPPC lipid bilayer. These findings provide detailed information on the effect of lupeol on the DPPC head group and the conformation and dynamics of the hydrophobic chains. In conclusion, the effect of lupeol on the structural features of the DPPC membrane, specifically phase transition and lipid packing, has implications for understanding its biological function and its applications in biotechnology and medicine.

## Introduction

The worldwide population is getting older, and cancer is often regarded as being one of the leading causes of death in the world. The development of new drug-targeted therapy will surely reduce the occurrence of cancer in the next few years. Nevertheless, the incidence of chronic diseases like cancer will keep rising. Thus, the quest for a more secure and affordable treatment is of critical importance (Sheikh et al. 2021). Recent work in the development and research of anticancer therapies that use natural products has resulted in the discovery of several terpenoids that inhibit cancer cell proliferation and metastasis through a variety of methods (Huang et al. 2012).

Terpenoids are the most abundant class of natural chemicals, serving as an enormous repository of potential therapeutic candidates. Terpenoids are classified into five subclasses depending on their structures: monoterpenoids, sesquiterpenoids, diterpenoids, triterpenoids, and tetraterpenoids (Huang et al. 2012). Much evidence has shown that the pentacyclic triterpenoids of the oleanane, ursane, lupane, and friedelane kinds (oleanolic, ursolic, betulinic, 18-glycyrrhetinic, asiatic acids, celastrol, lupeol, among others) have anti-cancer activities. These substances cause apoptosis in a variety of cancer cell types, including skin, breast, colon, and prostate tumor cells as well as inhibit tumor development and survival (Proshkina et al. 2020).

**Fig. 1:**
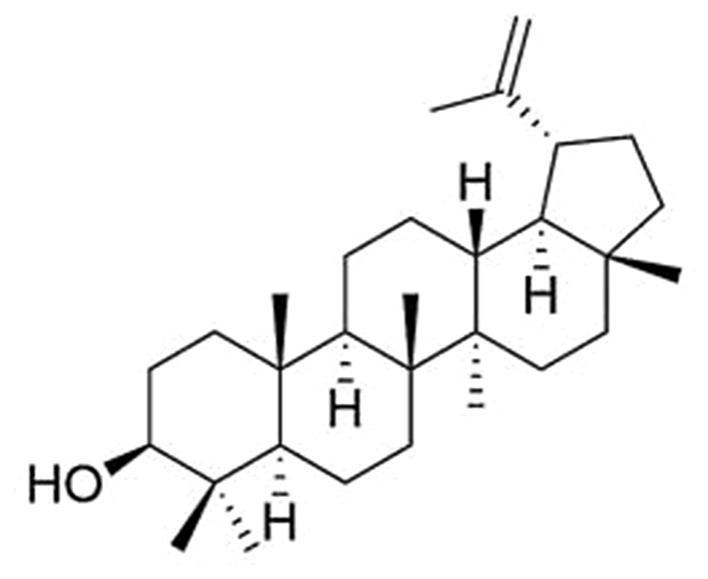
Chemical structure of lupeol (**A**)

Lupeol ((3-beta)-Lup-20(29)-en-3-ol) (Fig. 1) is a pentacyclic triterpenoid that has captured the attention of medical practitioners, researchers, and pharmaceutical marketers due to its diverse pharmacological properties (Siddique and Saleem 2011). Lupeol’s chemical formula is C_30_H_50_O, with a melting point of 215–216 °C and a molecular weight of 426.7174 (g/mol). Lupeol’s infrared spectrum reveals the existence of a hydroxyl function and an olefinic moiety at 3235 and 1640 cm^− 1^, respectively (Sharma et al. 2020). Lupeol is present in a variety of plants and fruits, including tomato, white cabbage, cucumber, carrot, pea, pepper, bitter root, soybean, black tea, guava, strawberries, ivy gourd, red grapes, mulberries, figs, and date palm (Siddique and Saleem 2011). It is also frequently detected in plants such as *Crataeva nurvala* (Buch Ham), *Betula platyphylla*, the roots of *Anemone raddeana, Hieracium pilosella*, the bark of *Gossampinus malabarica, Tamarindus indica*, *Arbutus unedo*, *Tipuana tipu*, the latex of *Leptadenia hastate*, and *Acacia mellifera* (Chaturvedi et al. 2008). Several in vitro and preclinical animal investigations indicate that lupeol has the potential to serve as an antimicrobial, anti-protozoal, anti-invasive, anti-inflammatory, anti-proliferative, anti-angiogenic, and cholesterol-lowering agent (Wal et al. 2015). Lupeol has been shown to have extremely low toxicity levels. Lupeol taken orally at a dose of 2 g/kg body weight has been shown to have no deleterious effects on rats and mice, with no mortality after 96 h of observation (Chaturvedi et al. 2008). Additionally, lupeol reduces oxidative stress in the tissues of the eyes and helps rats with cataracts caused by selenite (Proshkina et al. 2020).

Membranes that are as dynamic and complex as the cell itself define the boundaries of biological cells and organelles. The cell membrane is a complex mixture of proteins, lipids, and carbohydrates that performs a variety of tasks, such as enzymatic activity, receptor binding, molecularly specialized transport, and regulation of cell-cell interactions (Plant 1999). Due to the fact that biological membranes are too complicated to accurately characterize, analytical research employs simplified model systems (Hasan and Mechler 2017). Biomimetic lipid membranes have yielded detailed information on a variety of topics, including lipid phase transitions, bilayer structure, the effect of cholesterol on the structure and dynamics of lipid bilayers, and interactions with drugs, peptides, and proteins (Luchini and Vitiello 2021). Phosphatidylcholines (PC), the most prevalent phospholipids found in biomembranes, are usually the basis of model systems. PC is commonly found in neat, binary, or ternary mixes (Hasan and Mechler 2017).

Terpenoids’ biological activities have been studied extensively, but their molecular mechanisms remain unclear and in the literature, few publications exist on their membrane interactions. For example, Prades et al. (Prades et al. 2011) investigated the effects of oleanolic, maslinic, and ursolic as the most important free triterpenic acids found in orujo olive oil on phospholipid membranes. Different physicochemical techniques, such as X-ray diffraction (XRD), differential scanning calorimetry (DSC), ^31^P nuclear magnetic resonance (NMR), and Laurdan fluorescence were used. Experimental data show that all three triterpenic acids change the structural properties of DPPC and DPPC-Chol (cholesterol) membranes. Calorimetric data on the effects of all three compounds on DPPC and POPC (1-palmitoyl-2-oleoyl sn-glycero-3-phosphocholine) membranes were considered in detail. It was observed that the pretransition temperature was not affected much depending on the concentration. The triterpenic acids caused the main phase transition peak to broaden, separate into two peak components, and decrease the main phase transition enthalpy at high concentrations. The DSC results of these three compounds with POPC bilayers also showed a similar profile to the results obtained with DPPC bilayers. It was determined that these compounds form two- or three-component phase transition peaks in cholesterol-enriched DPPC bilayers (DPPC: Chol 70:30), and thus these peaks may consist of multiple coexisting domains.

Lőrincz et al. (Lorincz et al. 2015) researched the influences of ursolic acid (UA), a plant-derived triterpenic molecule, on the structural and morphological characteristics of DPPC-water systems by using DSC, small and wide-angle X-ray scattering (SWAXS), freeze-fracture transmission electron microscopy (FF-TEM), and Fourier transformation infrared spectroscopy (FTIR) techniques. According to the DSC results, it was found that ursolic acid at a molar concentration of UA/DPPC = 0.01 caused a very small perturbation in the thermal behavior of the system. Domains rich in ursolic acid molecules present as a broad shoulder on the right side of the main phase transition, and this occurs more dominantly at increasing ursolic acid concentrations (UA/DPPC = 0.1, 0.2, and 0.3). Domains rich in ursolic acid show thermodynamically stable formations due to the shift of the main transition to the positive side. In the FTIR spectroscopy results, it was observed that a new band was formed in the region of approximately 1691 cm^–1^ due to the presence of ursolic acid in the system. It is thought that this new small band observed at UA/DPPC = 0.1, 0.2 and 0.3 molar ratios may result from the aggregation of a part of ursolic acid in dimer form. The temperature dependence of the wavenumber of the CH_2_ symmetric stretching mode of ursolic acid-DPPC liposomes is examined and it was determined that the wavenumber values of the DPPC system containing different ursolic acids in the gel phase shifted towards high values, whereas in the liquid crystal phase they remained at low wavenumber values.

Abboud et al. (Abboud et al. 2016) examined the effect of tetracyclic (cortisol, prednisolone, and 9-fluorocortisol acetate) and pentacyclic (uvaol and erythrodiol) triterpenes (TTPs) on the fluidity of DPPC membranes in detail by DSC, Raman spectroscopy, and fluorescence anisotropy. According to the DSC results, in liposomes containing various molar concentrations (DPPC: TTP = 100:1, 100:2.5, and 100:10) of cortisol (Co), prednisolone (PD), and 9-fluorocortisol acetate (9-FA), the main phase transition temperature shifts towards lower values, and the pretransition completely disappears. A shoulder occurred in the main phase transition of prednisolone and 9-fluorocortisol acetate at the concentration of DPPC: TTP = 100:10. From the Raman spectroscopy results, the presence of these compounds in the DPPC membrane system indicates an increase in the I_2935/2880_ intensity ratio, and therefore an increase in rotational disorders. It is seen that these compounds cause an increase in the I_2844/2880_ intensity ratio. It has been observed that the tetracyclic and pentacyclic triterpenes used increase the *gauche/trans* ratio and disrupt the lipid chain order, as the height intensity ratios of the peaks observed in the 1000–1200 cm^–1^ region allow direct comparison of order-disordered transitions between I_1090/1130_ liposome samples. Since the peak at 715 cm^–1^ observed in the 700–800 cm^–1^ region corresponds to the stretching vibration of the C–N band of the choline group of DPPC, it was determined that these triterpenes significantly reduced the intensity of the observed peak.

In this study, we investigated the effect of lupeol on 1,2‑dipalmitoyl‑sn‑glycerol‑3‑phosphocholine (DPPC) model membranes by using differential scanning calorimetry (DSC) and Fourier transform infrared (FTIR) spectroscopy. We chose DPPC and lupeol for two main reasons: (i) phosphatidylcholines (PC) are among the most abundant phospholipids found in mammalian cell membranes (ca. 45–55%), providing a simple model of a lipophilic environment to mimic cell membranes, even though DPPC itself is mainly present in lung and brain tissues, (ii) the mechanism of action of lupeol has not yet been elucidated in detail. The most popular calorimetric technique for examining the thermotropic phase behavior of liposomes and tracking the impact of host molecules on the gel-to-liquid crystalline acyl chain melting transition is differential scanning calorimetry. FTIR spectroscopy can quickly and label-freely produce a chemical fingerprint of the sample being studied. This vibrational technique, in particular, makes it possible to gather information on the structure and composition of complex biological systems, such as entire cells and tissues, in addition to the major isolated biomolecules (Mereghetti et al. 2014). DSC and FTIR data can be combined to study the type of interaction, depth of penetration in the bilayer, and conformational changes in the hydrophobic chain structure (Bonora et al. 2003). These interactions also affect the partitioning, direction, and conformation of terpenoids in bilayers, making them crucial for lipidic drug delivery systems (Mady et al. 2012).

## Materials and methods

### Chemicals

Lupeol, phosphate buffered saline (PBS), chloroform, and ethanol were purchased from Sigma-Aldrich (St. Louis, MO, USA). DPPC (1,2-dipalmitoyl-sn-glycero-3-phosphocholine) was obtained from Avanti Polar Lipids (Alabaster, AL, USA) and stored at − 20 °C.

### Preparation of liposomes

Liposomes were prepared using the Bangham method (Bangham et al. 1965; Bangham 1978). DPPC and lupeol were dissolved in chloroform and ethanol, respectively. For calorimetric and spectroscopic experiments, the amounts of DPPC were used according to the values determined by Altunayar-Unsalan et al. (Altunayar-Unsalan et al. 2022a). The relative molar concentrations of lupeol were varied (i.e., 1, 5, 10, and 20 mol%). The mixtures were evaporated under a stream of nitrogen and then the samples were kept in a high vacuum for about 2 h to form thin, homogeneous, solvent-free films. In order to create liposomes, the dry lipid-lupeol films were hydrated with 10 mM PBS buffer in deionized water at pH 7.4 at temperatures that were approximately 20 °C above the main lipid phase transition and vortexed for 20 min.

### Differential scanning calorimetry (DSC)

Differential scanning calorimetry measurements were performed using TA Q20 DSC (TA Instruments Inc., New Castle, Delaware, USA) instrument with a heating rate of 1 °C/min in a temperature interval of 5 to 50 °C. Experimental runs were carried out under a dry nitrogen atmosphere with a gas flow of 50 mL/min. Samples were encapsulated in standard aluminum DSC pans. The DSC data were analyzed using Universal V4.5 A TA Instruments software. DSC records a sample’s temperature-dependent isobaric heat capacity, *C*_*p*_*(T).* Considering first-order transitions like gel-to-liquid crystalline transition of the lipid bilayer, the main phase transition temperature T_m_ is the temperature at which the heat capacity reaches its maximum. Integrating the area under the peak yields the value of calorimetric enthalpy ($\varDelta {H}_{c}$) for the main phase transition (Raudino et al. 2011):

$$\varDelta {H}_{c}= \int {C}_{p}dT$$

### Attenuated total reflection Fourier transform infrared (ATR-FTIR) spectroscopy

Infrared spectroscopic measurements of liposomes with and without lupeol were performed using a Fourier transform infrared spectrometer equipped with an attenuated total reflection set-up. ATR-FTIR spectra were recorded with a PerkinElmer Spectrum Two FTIR spectrometer (PerkinElmer Inc., Waltham, MA, USA) equipped with a deuterated triglycine sulfate (DTGS) detector and a diamond crystal as an internal reflection element. All spectra were taken in the scan range of 4000–1000 cm^–1^ with an average of 64 scans and a spectral resolution of 2 cm^–1^. The data interval for the wavenumbers in the IR spectra was 0.5 cm^–1^. Before the measurements, the ATR crystal was carefully cleaned with ultra-pure organic solvents, and a clean crystal was used as a background. All experiments were carried out at ambient temperatures. The spectra were analyzed using PerkinElmer Spectrum v10.5.4 software (PerkinElmer Inc., Waltham, MA, USA) and corrected for water vapor and CO_2_ contributions. OH stretching bands that arise from the PBS buffer are observed in the 3400–3200 cm^–1^ and 1800–1500 cm^–1^ regions. In addition, these bands overlap with the corresponding bands of lipids. For this reason, the buffer spectrum was recorded and then subtracted from the spectra of liposomes. Since the buffer spectrum overlaps with the spectra of liposomes, a manual subtraction procedure was applied using PerkinElmer Spectrum v10.5.4 software (PerkinElmer Inc., Waltham, MA, USA) until a proper baseline was achieved in the bulk water area, which is centered approximately 2100 cm^–1^. The subtracted original spectra were used to perform comprehensive analyses. In these analyses, the peak positions were measured with respect to the center of the weight, while the peak widths were determined from 80% of the peak’s highest position. Moreover, a normalization process was applied with regards to the relevant bands in order to clearly illustrate the spectral alterations in the spectra.

## Results and discussion

### Differential scanning calorimetry (DSC)

Physical chemistry and biophysics are interested in the thermodynamic phase behavior of hydrated lipids because the physical and thermodynamic states of membranes are critical to their biological function (Hasan and Mechler 2017). Since phosphatidylcholines (PCs) and sphingomyelins are the main lipid components of cell plasma membranes, dipalmitoylphosphatidylcholine (DPPC) bilayers are frequently utilized as basic model membranes. Moreover, they have well-characterized structural and thermodynamic properties, which is a necessary precondition for researching how bioactive chemicals disturb membranes. It is anticipated that any interactions between bioactive chemicals and the bilayer can modify the thermal and structural characteristics of DPPC (Bourgaux and Couvreur 2014). Since DPPC exhibits a variety of phase transitions, four phases—liquid crystalline (L_α_), ripple (P_β′_), gel (L_β′_), and crystal subgel (L_c_)—are identified in decreasing temperature order, with the ripple phase being particularly difficult to identify. Water inclusion between DPPC molecules is often considered to be connected to the integrity of the layer (Moreira et al. 2019).

**Fig. 2:**
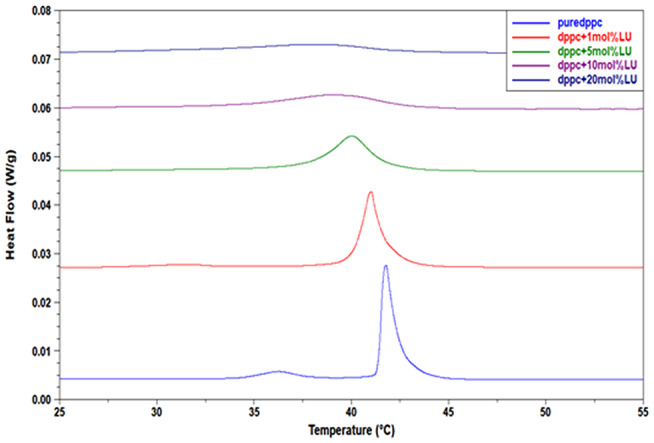
DSC thermograms of DPPC lipid bilayers with varying amounts of lupeol

**Fig. 3:**
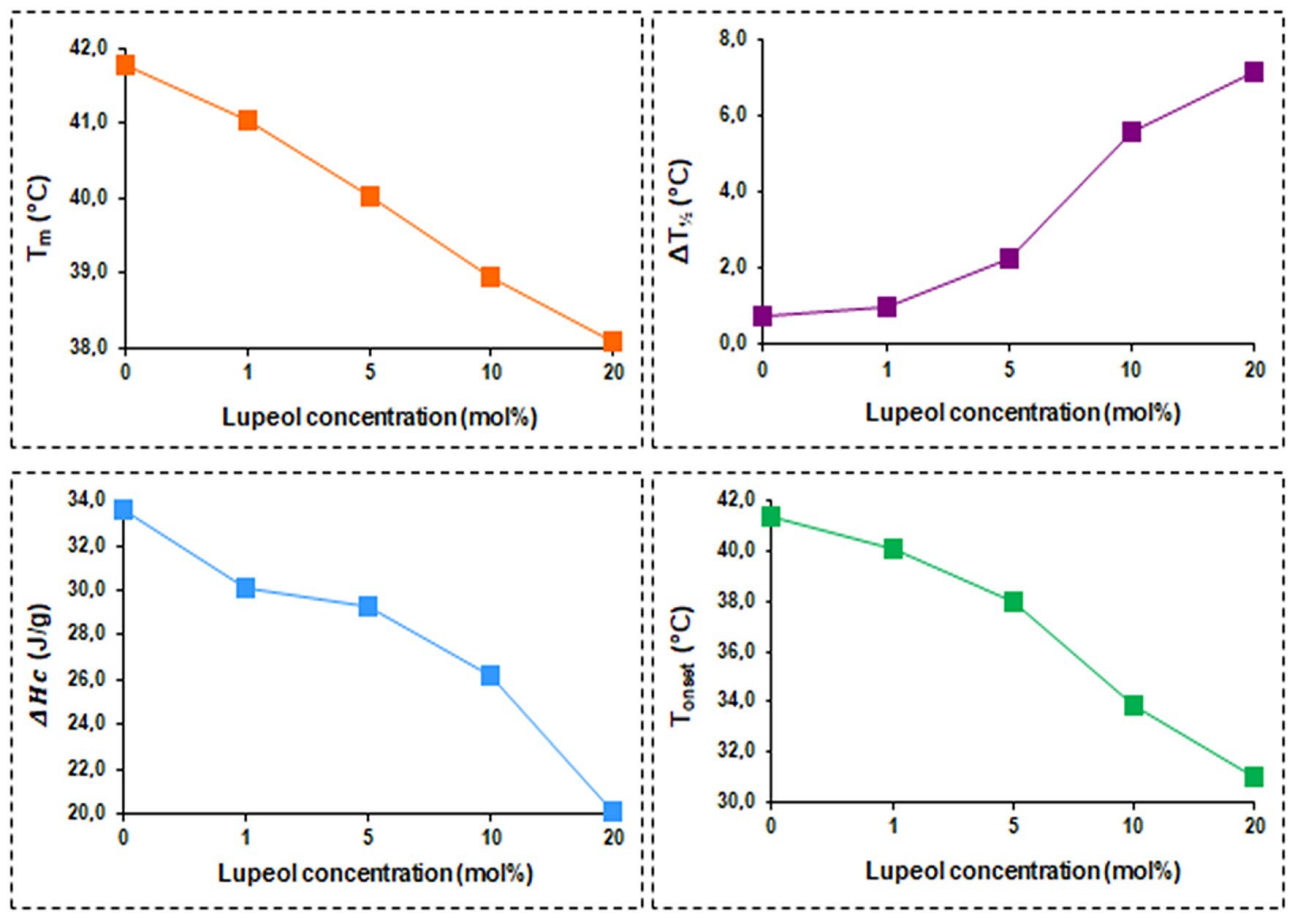
Main phase transition temperatures (T_m_), full width at half maximum (ΔT_½_), calorimetric enthalpy (ΔH_c_), and onset temperatures (T_onset_) of the main phase transitions for lupeol containing DPPC liposomes

Figure 2 shows two distinct peaks on the pure DPPC thermogram located at 35.15 and 41.78 °C. The mobility of the choline polar head of DPPC is responsible for the low-enthalpy transition shown in the first peak, whereas the mobility of the alkyl chains is responsible for the sharp-enthalpy main transition (Gardikis et al. 2006). The main transition’s thermodynamic parameters—that is, the temperature at which heat flow reaches its maximum—were ascertained: T_m_ = 41.78 °C, the transition’s cooperativity indicated by the peak width at half-height: ΔT_1/2_ = 0.73 °C, and the transition’s enthalpy found from the area under the DSC curve: ΔH_c_ = 33.48 J/g. These results are consistent with information published in the literature (Biltonen and Lichtenberg 1993; Tahir et al. 1999). Figure 2 also displays the DSC scans for the lupeol/DPPC mixtures. Several concentration-dependent actions are induced by lupeol’s presence in the DPPC lipid bilayer. The inclusion of lupeol at a low concentration (5 mol%) eliminates the pretransition peak at 35.15 °C. It is possible that the interactions between the DPPC head groups and the lupeol polar groups eliminate this low-enthalpy transition. The DPPC main transition broadens in the presence of a low concentration of lupeol, which may indicate a decrease in cooperativity between the acyl chains of DPPC. The greater the amount of lupeol, the more the main transition peak (i) shifts to lower temperatures, (ii) broadens continually, and (iii) reduces in intensity. Using DSC thermograms, the thermodynamic parameters of the main transition—T_m_, ΔT_1/2_, ΔH_c_, and T_onset_ —are plotted against the concentration of lupeol (Fig. 3). As the concentration of lupeol increases, T_m,_ T_onset_ and ΔH_c_ decrease, whereas ΔT_1/2_ increases. Thus, more fluid bilayers are produced when the van der Waals forces between the hydrocarbon chains are weakened by the lupeol molecules that are incorporated. It may be deduced that the lipid bilayer becomes heterogeneous at high-incorporated lupeol concentrations (low ΔH_c_ values) because these weak van der Waals interactions control the membrane’s structure.

### ATR-FTIR spectroscopy

**Fig. 4:**
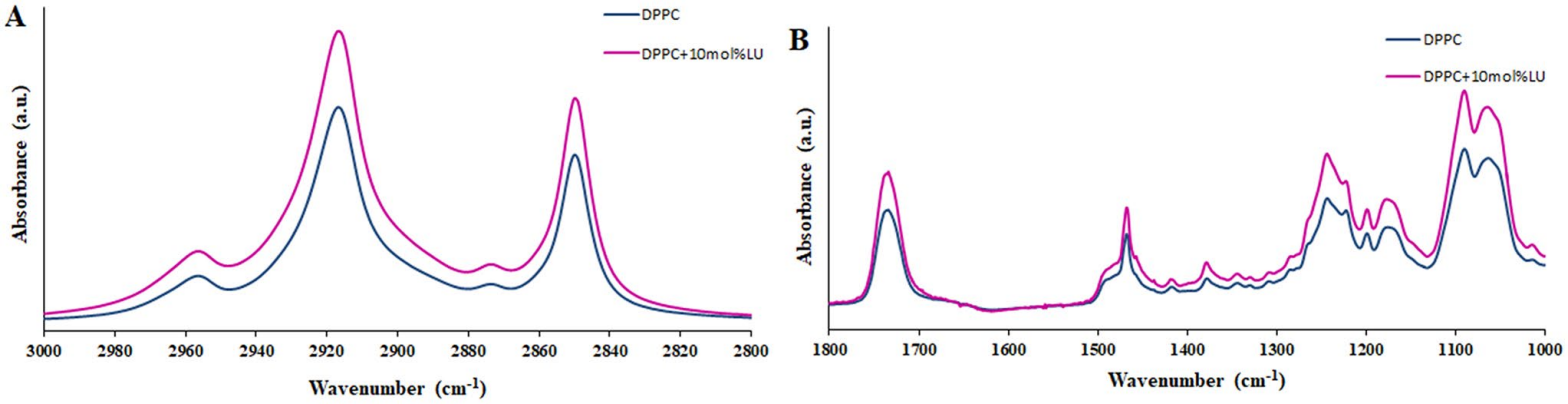
FTIR spectra of DPPC liposomes without and with lupeol. (A) 3000 –2800 cm^‒1^, (B) 1800 –1000 cm^‒1^

Using ATR-FTIR spectroscopy, which is sensitive to changes in lipid conformation, the impact of lupeol binding on DPPC lipid bilayers is investigated with respect to hydrocarbon chain organization and hydration of the lipid head groups. Figure 4 displays the infrared absorption spectra of two DPPC membranes, one pure and the other modified with lupeol. In the pure DPPC spectrum, the C-H stretching modes are represented as the major band between 2800 and 3000 cm^− 1^, with the maxima of peaks at 2848 and 2916 cm^− 1^, respectively, corresponding to the symmetric and antisymmetric stretching in the alkyl chain’s CH_2_ groups with the symmetric and antisymmetric stretching vibrations in the CH_3_ groups contributing insignificantly at 2873 cm^− 1^ and 2955 cm^− 1^, respectively (Mady and Elshemey 2011; Pawlikowska-Pawlȩga et al. 2012). Lupeol binding affects the hydrophobic core area of membranes, as shown by the location of the antisymmetric or symmetric CH_2_ stretching vibration (ʋ_as_(CH_2_) or ʋ_s_(CH_2_). It is common to interpret a decrease in the ʋ_as, s_(CH_2_) wavenumber as an increase in the ordering of the lipid acyl chains (Hoernke et al. 2012). Bandwidth in the same bands provides dynamic information regarding the system (Zhao and Feng 2006). In addition, the strong band at 1735 cm^− 1^ represents the stretching vibrations of DPPC’s ester carbonyl groups (Pawlikowska-Pawlȩga et al. 2012). Changes in the hydration of the lipid head group area can be detected by the wavenumber of the lipid ester carbonyl stretching vibration. The number and strength of H-bonds at the C = O group determine this band’s wavenumber (Hoernke et al. 2012). Furthermore, the PO_2_^−^ antisymmetric stretching mode at 1246 cm^− 1^ and the symmetric stretching mode at 1090 cm^− 1^ are crucial bands for studying DPPC interactions with lupeol at the head group region (Arczewska et al. 2013).

**Fig. 5:**
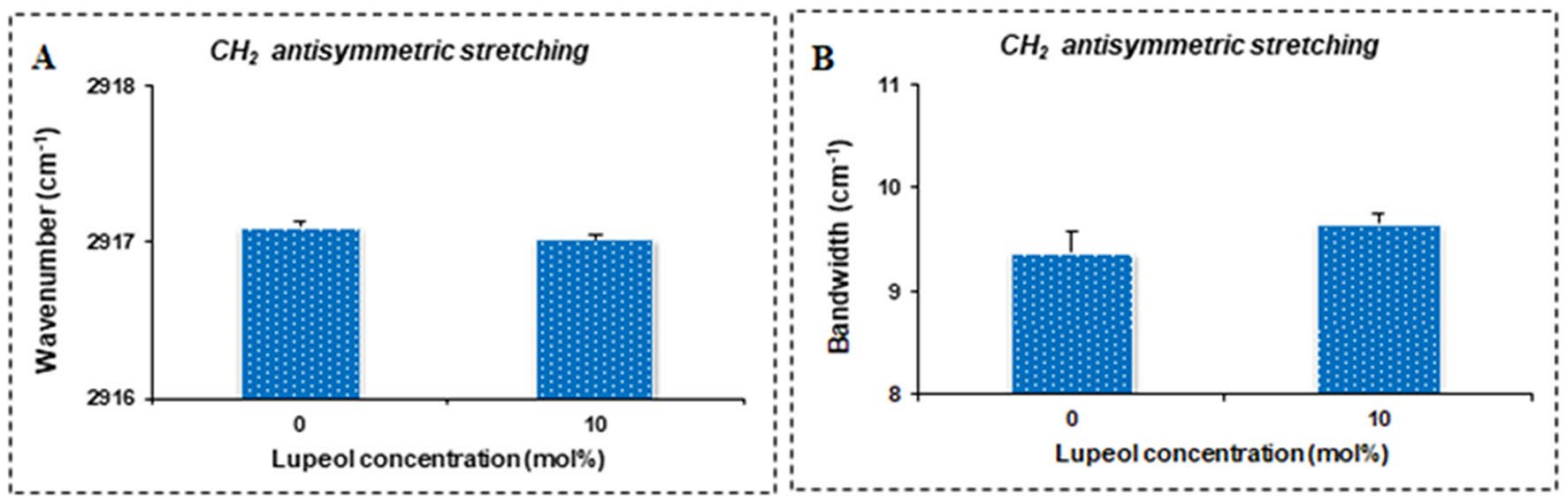
Lupeol concentration dependence of the wavenumber of the CH_2_ antisymmetric stretching mode of DPPC liposomes (**A**). Lupeol concentration dependence of the bandwidth of the CH_2_ antisymmetric stretching mode of DPPC liposomes (**B**)

Figure 5A shows the lupeol concentration dependence of the wavenumber of the CH_2_ antisymmetric stretching mode of DPPC liposomes. 0 mol% indicates pure DPPC liposomes without lupeol. The addition of 10 mol% concentration of lupeol into pure DPPC liposomes causes a shift of the wavenumber to lower values, reflecting an increase in the number of *trans* conformers and a more ordered state (Altunayar-Unsalan et al. 2022a, b). Lupeol concentration dependence of the bandwidth of the CH_2_ antisymmetric stretching mode of DPPC liposomes is given in Fig. 5B. Adding 10 mol% lupeol to pure DPPC liposomes increases the bandwidth values of the CH_2_ antisymmetric stretching band, indicating an increase in the mobility of acyl chains (Altunayar-Unsalan et al. 2022a, b).

**Fig. 6:**
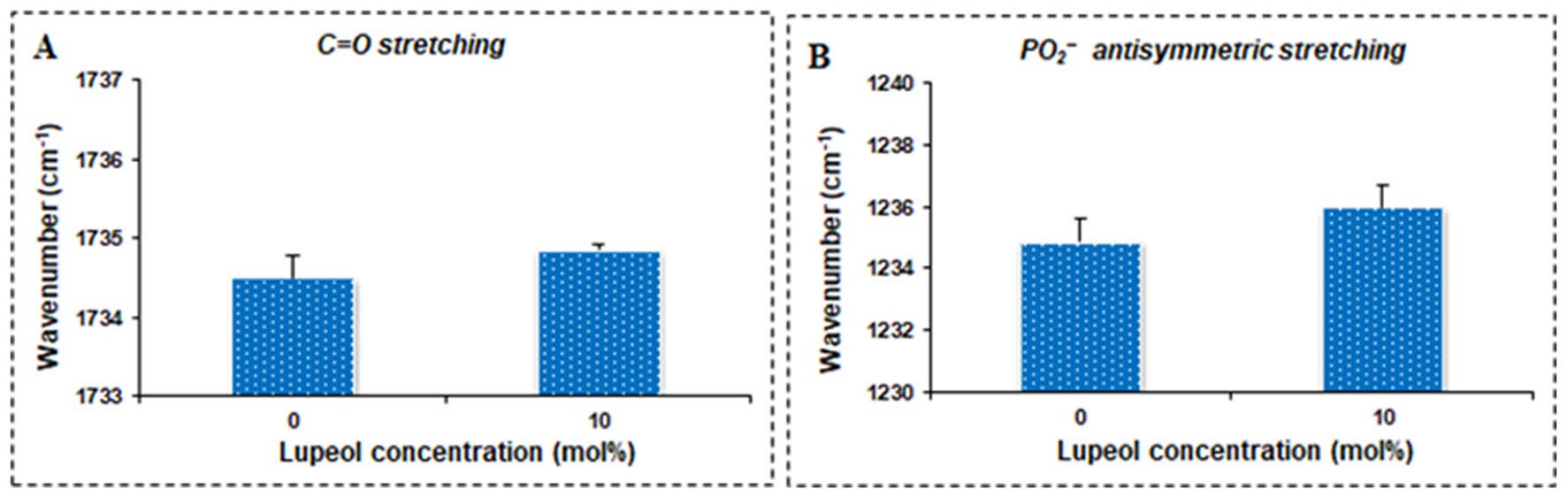
Lupeol concentration dependence of the wavenumber of the C = O stretching mode of DPPC liposomes (**A**). Lupeol concentration dependence of the wavenumber of the PO_2_^−^ antisymmetric stretching mode of DPPC liposomes (**B**)

Figure 6A shows the dependence of lupeol concentration on the wavenumber of the C = O stretching mode of DPPC liposomes. Pure DPPC liposomes contain 0 mol% lupeol. As shown in Fig. 6A, the wavenumber of the C = O stretching band shifts to higher values with the incorporation of 10 mol% of lupeol into pure DPPC liposomes, indicating that lupeol displays nearly identical patterns of dehydration in the glycerol-acyl chain interface of DPPC liposomes (Altunayar-Unsalan et al. 2022a, b). Lupeol concentration dependence of the wavenumber of the PO_2_^−^ antisymmetric stretching mode of DPPC liposomes is shown in Fig. 5B. As shown in Fig. 6B, the presence of 10 mol% of lupeol produces a shift in the wavenumber to higher values, resulting in dehydration in the polar head group of DPPC liposomes (Altunayar-Unsalan et al. 2022a, b).

In this study, the interaction of lupeol, a molecule in the lupane group of the triterpenoid family, with DPPC model membrane lipid systems is examined using calorimetric and spectroscopic methods. These investigations, together with the use of experimental biophysical methods such as DSC and ATR-FTIR spectroscopy and temperature and concentration-dependent measurements, allow us to expand the research on terpenes and their derivatives, terpenoids, and lipid model membrane systems in the literature and to obtain more detailed and in-depth information about the interactions between terpenes/terpenoids and membrane lipids. Although DPPC is a zwitterionic phospholipid, that is, it contains both positive and negative head groups, very small changes in the molecular structures of the lupeol molecule affect the interaction of this molecule with the head group of DPPC and, therefore its position in the membrane.

While many studies have been conducted on lupeol’s biological activities (Zhang et al. 2019, 2021; Efimova et al. 2022), the molecular mechanisms responsible for these activities remain unclear. Additionally, there are few publications on the nature of lupeol’s membrane interactions. Broniatowski et al. (Broniatowski et al. 2012) investigated the interactions of lupeol (LU) and betulinic acid (BA) with saturated glycerophosphatidylcholine (DPPC) and stearoyl-sphingomyelin (SM) using the Langmuir technique, Brewster angle microscopy and grazing incidence X-ray diffraction to confirm findings obtained on living cellular lines in model systems. Four binary monolayer systems (LU/DPPC, BA/DPPC, LU/SM and BA/SM) were examined as follows: X(terpenes) = 0.2, 0.33, 0.5, 0.67, 0.8, 0.94. Consider the systems LU/DPPC and LU/SM, as X(LU) values increased, collapse pressures decreased.

The decrease in collapse pressure with X(LU) indicated mutual miscibility of the components in a binary film. In the system LU/DPPC for X(LU) < 0.66, several tiny domains were present in the monolayer. The film had the long worm-like or X-shaped domains beginning with X(LU) = 0.66. All of the images for this system showed that the binary mixes of lupeol and DPPC have distinct textures than the films of pure component. In the system LU/SM, the mixtures with X(LU) < 0.66 were totally homogenous, whilst the worm-like and X-shaped domains exhibited at higher X(LU) diverged drastically from the textures observed in pure SM monolayers.

Rodríguez et al. (Rodríguez et al. 1997) studied the effect of several pentacyclic triterpenes (PTs) on the structural and dynamic properties of DPPC bilayers by fluorescence measurements. The presence of 12 mol% concentration of lupeol resulted in a higher decrease in the r_S_ (steady-state fluorescence anisotropy) of two probes DPH (1,6-diphenyl-1,3-5-hexatriene) and TMA-DPH (1-(4-trimethyl-ammoniumphenyl)-6-phenyl-1,3,5-hexatriene) than that of cholesterol for the gel state, however it only slightly increased in the r_S_ for the liquid–crystalline state. In addition, lupeol at 12 mol% had no influence on the ordering of the liquid-crystalline state as detected by both probes. In the gel state, 12 mol% lupeol lowered r_∞_ (limiting anisotropy) of both probes. Thus, it was determined that pentacyclic monoalcohols are more active than cholesterol in lowering the DPPC gel phase order but less effective in increasing the liquid-crystalline state order. Finally, the pentacyclic triterpenes may be oriented similarly to cholesterol in the bilayer with their 3-*β*-OH toward the outside. Lupeol is present in smaller amounts, indicating it does not interact well with the bilayer structure. Lupeol possesses geometric properties that could cause the bilayer structure to be unstable. In lupeol, the isopropenyl group linked to C-19α increases the molecular dimensions of the E ring. These structural characteristics may explain lupeol’s poor incorporation levels in DPPC bilayers.

## Conclusion

Lupeol’s interaction with the DPPC membrane is studied using non-invasive techniques, DSC and FTIR spectroscopy. Based on the results obtained in the present study, we propose that lupeol can be readily integrated into DPPC lipid bilayers, influencing their thermotropic phase behavior, FTIR spectra, membrane order, and fluidity. Lupeol interacts with the DPPC’s hydrophilic and hydrophobic groups, which are observed as spectral changes in the bands corresponding to CH_2_ antisymmetric stretching, C = O stretching, and PO_2_^−^ antisymmetric stretching vibrations. These findings contribute to a better knowledge of the interactions between lupeol and DPPC lipid bilayers, which could aid in the design and development of enhanced lipidic drug delivery systems.

## Data Availability

No datasets were generated or analysed during the current study.
